# Serum IgE in the clinical features and disease outcomes of IgG4-related disease: a large retrospective cohort study

**DOI:** 10.1186/s13075-020-02338-1

**Published:** 2020-10-23

**Authors:** Jiaxin Zhou, Yu Peng, Linyi Peng, Di Wu, Jing Li, Nan Jiang, Jieqiong Li, Hui Lu, Zheng Liu, Xuan Luo, Fei Teng, Yunyun Fei, Wen Zhang, Yan Zhao, Xiaofeng Zeng

**Affiliations:** 1 Department of Rheumatology, Peking Union Medical College Hospital, Chinese Academy of Medical Sciences, Peking Union Medical College, #1 Shuai-Fu-Yuan, Dongcheng District, Beijing, 100730 China; 2 National Clinical Research Center for Dermatologic and Immunologic Diseases (NCRC-DID), The Ministry of Education Key Laboratory, Beijing, China

**Keywords:** IgG4-related disease, Immunoglobulin E, Relapse

## Abstract

**Objective:**

The aim of this study was to investigate the role of serum IgE levels in the clinical features and outcomes of IgG4-related disease (IgG4-RD).

**Methods:**

We retrospectively enrolled 459 newly diagnosed IgG4-RD patients with serum IgE examined at baseline from 2012 to 2019 and compared the clinical features between group A (serum IgE level ≤ 60 KU/L) and group B (serum IgE level > 60 KU/L). Subsequently, 312 patients who had been followed up for ≥ 1 year were further selected to evaluate the correlation between serum IgE level and disease outcome.

**Results:**

At baseline, the serum IgE level was positively correlated with the serum IgG4 level (*r* = 0.1779, *P* = 0.0001), eosinophil count (*r* = 0.3004, *P* < 0.0001), and serum IgG level (*r* = 0.2189, *P* < 0.0001) in IgG4-RD patients. Compared with group A, group B had more patients with allergic diseases (*P* = 0.004), more organ involvement (*P* = 0.003), and higher IgG4-RD responder index scores (*P* = 0.002). During follow-up, group A patients had a higher remission induction rate than group B patients (88.4% vs. 73.6%, *P* = 0.035), while group B patients had a higher relapse rate than group A patients (29.0% vs. 16.2%, *P* = 0.039). Multivariate analysis found that a serum IgE level > 125 KU/L at baseline was a risk factor for disease relapse (hazard ratio [HR], 1.894 [95% confidence interval (CI) 1.022–3.508]; *P* = 0.042). Cox regression analysis showed that elevation of the eosinophil count was a risk factor for relapse in both group A and group B patients (HR, 8.504 [95% CI 1.071–42.511]; *P* = 0.009; and HR, 2.078 [95% CI 1.277–3.380]; *P* = 0.003, respectively), and the involvement of the lacrimal gland (HR, 1.756 [95% CI 1.108–2.782]; *P* = 0.017), submandibular gland (HR, 1.654 [95% CI 1.037–2.639]; *P* = 0.035), and kidney (HR, 3.413 [95% CI 1.076–10.831]; *P* = 0.037) were also risk factors for relapse in group B patients.

**Conclusion:**

IgG4-RD patients with high serum IgE levels at baseline were more likely to have higher disease activity, and baseline high IgE levels were associated with disease relapse.

## Introduction

IgG4-related disease (IgG4-RD) is a chronic fibroinflammatory disease that can affect nearly every organ system, has a distinctive histopathological pattern of IgG4-positive plasma cells that infiltrate the affected organs, and is usually accompanied by elevation of serum IgG4 levels [1, 2]. T follicular helper (Tfh) cells play an important role in the B cell production of both IgG4 and IgE, and elevated serum IgE levels are commonly observed in IgG4-RD patients [1, 3, 4]. Previous studies have shown that elevated baseline serum IgE levels might help diagnose and predict relapse of IgG4-RD [5–7]. However, there is still a limited understanding of the relationship between serum IgE and IgG4-RD. In this retrospective study, we conducted a thorough review of the clinical significance of serum IgE in both the clinical pattern and outcomes of IgG4-RD patients in a large cohort of Chinese patients.

## Patients and methods

### Patients’ enrollment

This retrospective study was based on a prospective cohort (registered on ClinicalTrials.gov; NCT01670695, study start date: January 2012), which was conducted at Peking Union Medical College Hospital (PUMCH) from 2012 to 2019. All patients fulfilled the 2019 American College of Rheumatology/European League Against Rheumatism Classification Criteria for IgG4-Related Disease [8, 9]. Patients with other autoimmune diseases, infectious diseases, or malignancies were excluded from this study. Patients’ history of allergic disease, including inhalation allergy, drug allergy, food allergy, contact allergy, and irritable physique, was collected using a thorough questionnaire at the baseline visit and was further confirmed by consultations from an allergist.

**Fig. 1:**
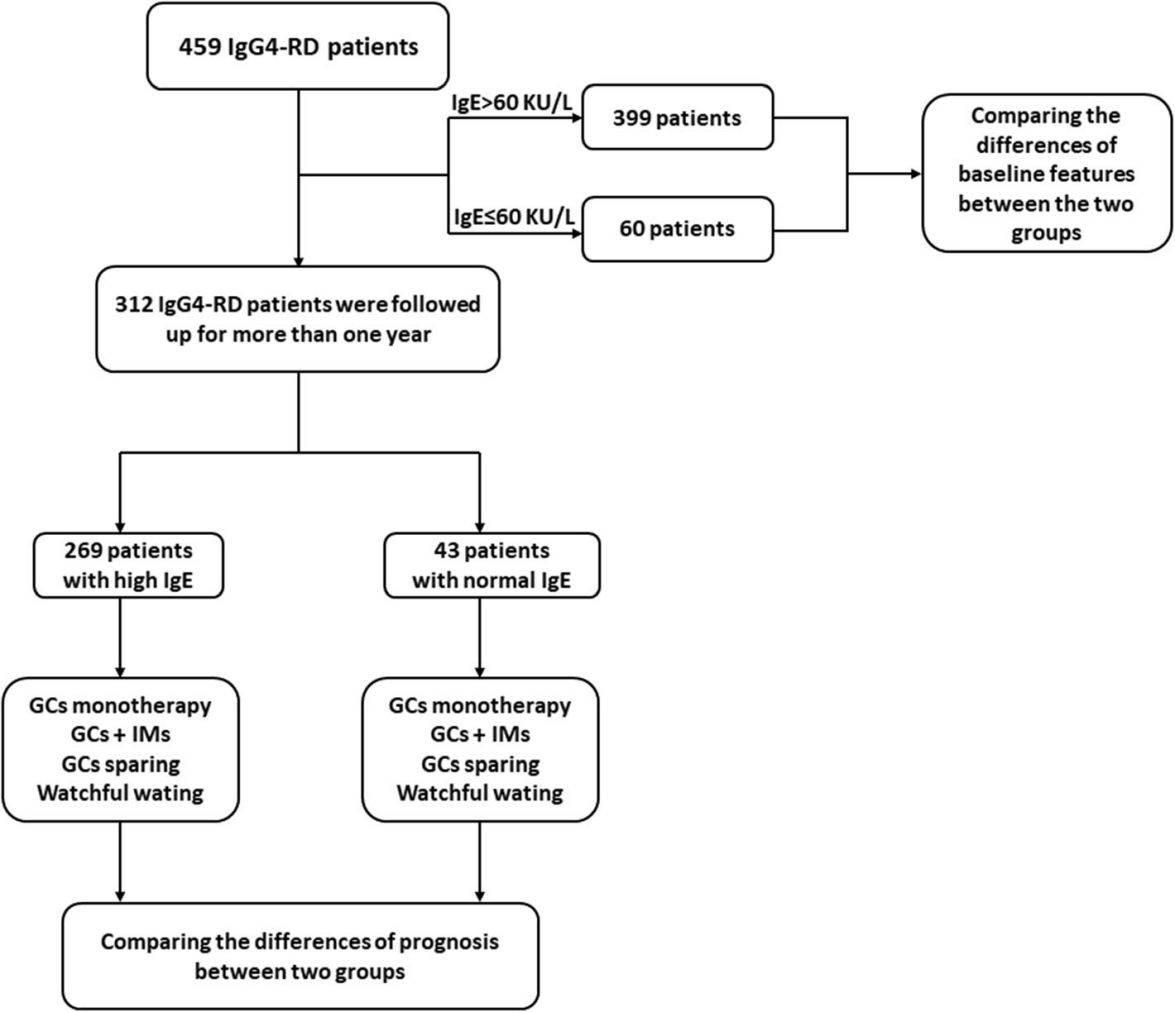
Diagram of the enrollment process of IgG4-RD patients

All newly diagnosed IgG4-RD patients with serum IgE examination at baseline were enrolled in this study. A serum IgE level > 60 KU/L was defined as elevated according to the range set by the institution. A total of 459 patients were enrolled in this study. Patients were subdivided into two groups: group A (*n* = 60) comprised those with serum IgE levels ≤ 60 KU/L, and group B (*n* = 399) comprised those with serum IgE levels > 60 KU/L. A total of 312 patients who were followed up for more than 1 year were selected for prognosis analysis. A diagram of the analysis procedure of enrolled patients is shown in Fig. 1.

### Laboratory tests, imaging studies, and histological examinations

Laboratory tests were conducted during enrollment and follow-up, including complete blood cell count, liver and renal function tests, erythrocyte sedimentation rates (ESR), C-reactive protein (CRP) levels, serum IgG and IgG subclass levels, serum IgE levels, complement 3 (C3) and complement 4 (C4) levels, rheumatic factor (RF), and antinuclear antibodies (ANAs). All patients underwent imaging examinations, such as ultrasound scanning (US), computed tomography (CT), magnetic resonance imaging (MRI), or positron emission tomography-computed tomography (PET-CT). Part of the patients with organ or tissue affected or swelling had a biopsy for further diagnosis of the disease.

### Treatment strategies

The treatment strategies used for the patients in this study could be divided into four types: glucocorticoid (GC) monotherapy, GCs combined with immunosuppressants (GCs + IMs), immunosuppressant monotherapy (GCs sparing), and watchful waiting. GCs were started with prednisone (or an equivalent dose) 0.6–0.8 mg kg^−1^d^−1^, continued for 2 weeks to 1 month, and then slowly reduced to the maintenance dosage with prednisone (or an equivalent dose) 5–10 mg d^−1^. The IMs in this study included mycophenolate mofetil (MMF), cyclophosphamide (CTX), azathioprine (AZA), methotrexate (MTX), cyclosporine A (CsA), leflunomide (LEF), and iguratimod.

### Assessment of disease activity and treatment response

Disease activity was assessed by the IgG4-RD responder index (RI) at each visit [10]. Patients’ treatment response can be evaluated through changes in their IgG4-RD RI scores. Disease relapse was defined as relapse or worsening of the clinical symptoms or imaging findings with or without re-elevation of serum IgG4 concentration [11]. Elevated serum IgG4 levels alone were not defined as disease relapse. More effective treatment strategies, including adding IMs and increasing the dosage of GCs and IMs, were added to all of the relapsed patients. Remission induction was defined in the first 6 months of the initial treatment, and patients who met the following conditions were thought to achieve remission induction: (1) IgG4-RD RI scores decreased by 50% or more than 50%; (2) GCs tapered to the maintenance dosage (prednisone ≤ 10 mg d^−1^ or equivalent dose); and (3) no relapse occurred during the GC tapering stage (first 6 months) [12].

### Statistical analyses

All patients’ data were collected and are listed in the tables of this article. Normally distributed quantitative variables are shown as mean ± SD, and the non-normally distributed features are presented as median (IQR). The comparison of the features between diverse groups was conducted using SPSS (version 22.0, IBM, Armonk, NY, USA) using the *t* test, chi-square test, Fisher’s exact test, and non-parametric test. A receiver operator characteristic (ROC) curve was used to determine the IgE cutoff value for disease relapse. Cox regression analysis was conducted in SPSS to determine the hazard ratio (HR) and the 95% confidence interval (CI) of the diverse features for groups A and B. *P* values < 0.05 were considered to indicate significant differences between the comparison groups.

## Results

### Baseline features of the patients enrolled in this study

**Fig. 2:**
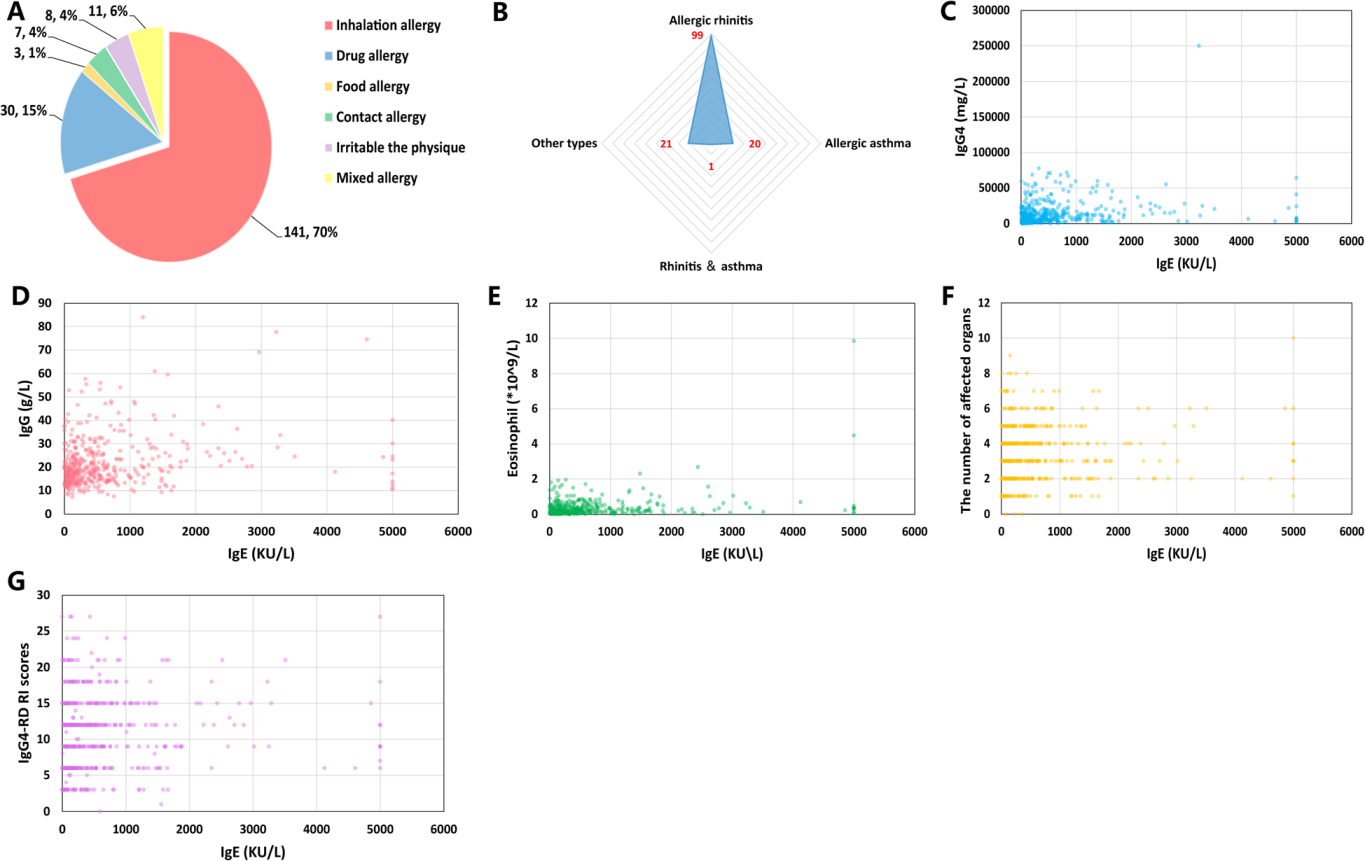
Composition of various allergies and correlations between IgE and other clinical features in patients with IgG4-related disease. **a** Distribution of allergy disease types in enrolled patients. **b** Statistics of the number of patients with different types of inhalation allergy. **c**–**g** Correlations between IgE and other clinical features of IgG4-RD patients at baseline (*P < 0.05 was thought to have statistical differences)

A total of 459 IgG4-RD patients were selected for this study, and the demographic and clinical features are shown in Table 1. The mean age at onset was 53.4 ± 12.8 years; male patients accounted for the majority with a gender ratio (male to female) of 1.7:1. It was noted that 201 (43.8%) patients had allergic diseases. Among them, patients with inhalation allergies (141/201, 70.1%) accounted for the majority, mostly allergic rhinitis (99/141, 70.2%), and 20 patients (20/141, 14.2%) had asthma (Fig. 2a, b). The overall prevalence of allergic rhinitis and asthma in our cohort was 21.6% (99/459) and 4.4% (20/459), respectively.

**Table 1:** Clinical features of the enrolled patients at baseline (*n* = 459) and during the follow-up period (*n* = 312)

	All enrolled patients (*n* = 459)	Patients who were followed up (*n* = 312)	*P* value
Demographic features			
Gender (male to female)	1.7:1	1.8:1	0.771
Age at onset (mean ± SD) (years)	53.4 ± 12.8	52.8 ± 12.4	0.519
Disease duration (months, median, IQR)	12 (4–36)	12 (4–36)	0.824
Allergy disease (*n*, %)	201 (43.8%)	150 (48.1%)	0.241
IgG4-RD RI scores (mean ± SD)	11.1 ± 5.3	11.7 ± 5.5	0.198
Follow-up period (months, median, IQR)	**–**	36 (24–54)	**–**
Organ involvement (*n*, %)			
Lacrimal gland	233 (50.8%)	166 (53.2%)	0.505
Parotid gland	73 (15.9%)	53 (17.0%)	0.690
Submandibular gland	239 (52.1%)	161 (51.6%)	0.899
Pancreas	166 (36.2%)	112 (35.9%)	0.939
Bile duct	86 (18.7%)	64 (20.5%)	0.541
Retroperitoneal tissue	79 (16.5%)	55 (17.6%)	0.881
Lung	117 (25.5%)	80 (25.6%)	0.962
Kidney	51 (11.1%)	34 (10.9%)	0.926
Lymph nodes	210 (45.8%)	153 (49.0%)	0.369
Liver	11 (2.4%)	7 (2.2%)	0.890
Paranasal sinus	130 (28.3%)	102 (32.7%)	0.194
Thyroid	13 (2.8%)	8 (2.7%)	0.822
Pituitary	7 (1.5%)	5 (1.6%)	0.932
Serological features (median, IQR)			
IgE (KU/L)	332 (125–756)	332 (146–699)	0.323
IgG4 (mg/L)	9202 (3580–18,325)	8960 (3730–16,600)	0.837
IgG (g/L)	18.83 (14.8–25.53)	18.65 (14.69–23.70)	0.678
Eosinophils (10^9^/L)	0.21 (0.11–0.43)	0.23 (0.12–0.48)	0.750
ESR (mm/h)	21 (8–50)	18 (7–44)	0.782
CRP (mg/L)	2.1 (0.76–6.9)	1.57 (0.63–5.3)	0.837
C3 (g/L)	0.93 (0.74–1.11)	0.94 (0.77–1.12)	0.981
C4 (g/L)	0.16 (0.11–0.23)	0.17 (0.11–0.22)	0.723

IQR stands for interquartile range. Patients’ data that did not meet the normal distribution are shown as median (IQR). SD stands for standard deviation, and the data meeting the normal distribution are shown as mean ± SD

The lacrimal gland, parotid gland, submandibular gland, lung, pancreas, lymph nodes, and paranasal sinuses were the most frequently affected organs of the IgG4-RD patients recruited in this study. As for the IgG4-RD-related serological parameters, serum IgE, IgG, and IgG4 were elevated. Through the Pearson correlation analysis, we found that serum IgE positively correlated with serum IgG4 levels (*r* = 0.1779, *P* = 0.0001), eosinophil count (*r* = 0.3004, *P* < 0.0001), and serum IgG levels (*r* = 0.2189, *P* < 0.0001) (Fig. 2c–g).

### Differences in clinical features between group A and group B

**Fig. 3:**
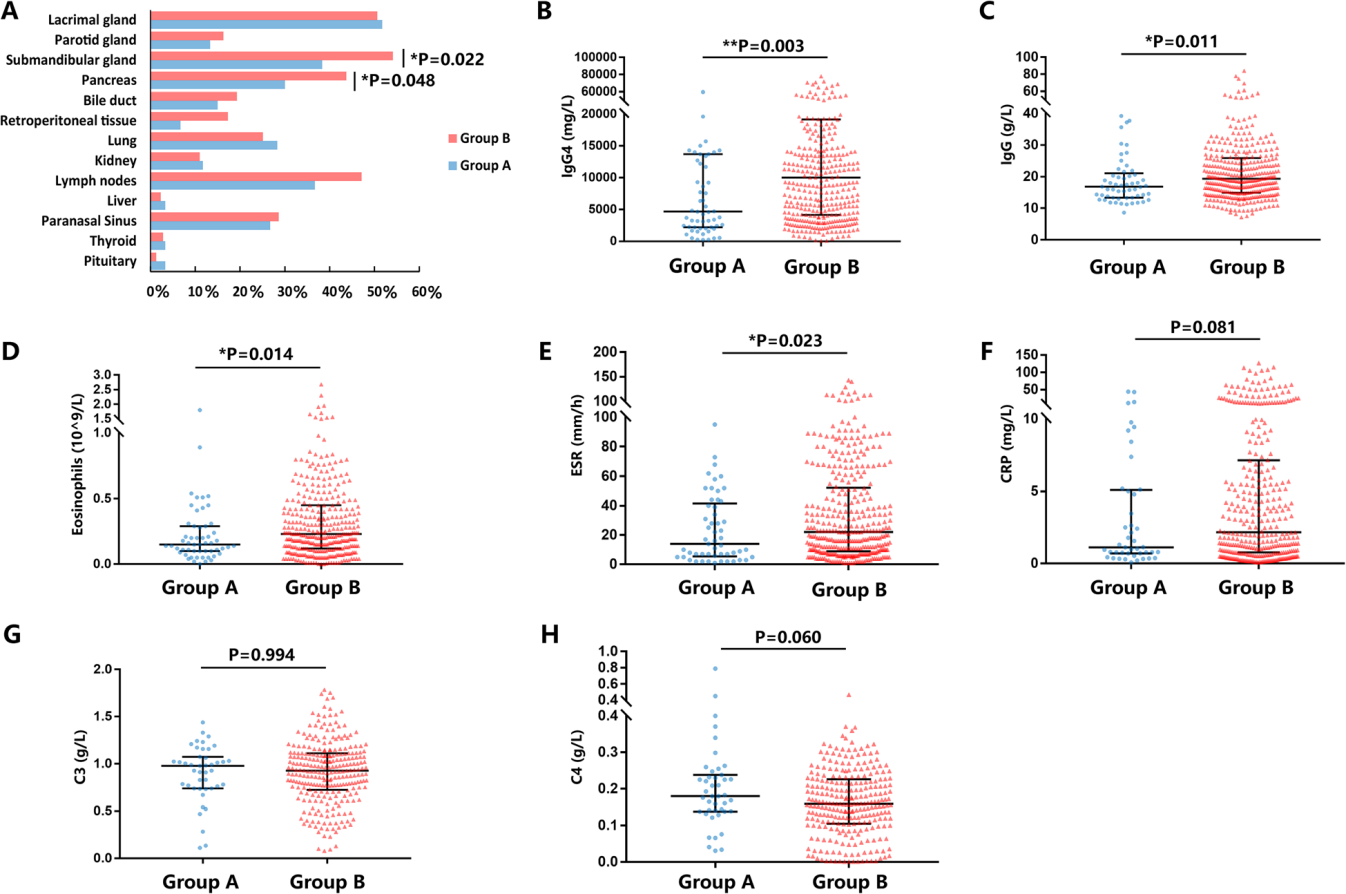
Comparison of the organ involvement and serological features between group A and group B patients. **a** Comparison of organ involvement between the two groups. **b**–**h** Comparison of serological features between the two groups (*P < 0.05, **P < 0.01, ***P < 0.001, ****P < 0.0001)

A comparison of the baseline clinical features between the two groups is shown in Table 2. Compared with group A, group B had more patients with allergic disease (*P* = 0.004), more organ involvement (*P* = 0.003), and higher IgG4-RD RI scores (*P* = 0.002). As for the difference in affected organs between the two groups, group B was more likely to have the submandibular gland (*P* = 0.022) and pancreas (*P* = 0.048) affected (Fig. 3a). Group B patients also had higher serum IgG4 levels (*P* = 0.003), higher serum IgG levels (*P* = 0.011), eosinophil counts (*P* = 0.014), and ESR (*P* = 0.023) at baseline (Fig. 3b–h).

**Table 2:** Comparison of baseline clinical features between group A (serum IgE level ≤ 60 KU/L) and group B (serum IgE level > 60 KU/L)

	Group A (*n* = 60)	Group B (*n* = 399)	*P* value
Gender (male, %)	39 (65.0%)	252 (63.2%)	0.782
Age at onset (mean ± SD)	51.2 ± 13.2	53.7 ± 12.8	0.152
Disease duration (months, median, IQR)	12 (4.25–36)	12 (4–36)	0.858
Allergic disease (*n*, %)	16 (26.7%)	185 (46.4%)	0.004**
Allergic disease family history (*n*, %)	7 (11.7%)	69 (17.3%)	0.274
Autoimmune disease family history (*n*, %)	4 (6.7%)	30 (7.5%)	0.814
Number of affected organs (median, IQR)	2 (2–4)	3 (2–5)	0.003**
IgG4-RD RI scores (mean ± SD)	9.3 ± 5.5	11.4 ± 5.2	0.002**
Organ involvement (*n*, %)			
Lacrimal gland	31 (51.7%)	202 (50.6%)	0.881
Parotid gland	8 (13.3%)	65 (16.3%)	0.559
Submandibular gland	23 (38.3%)	216 (54.1%)	0.022*
Pancreas	18 (30.0%)	148 (43.7%)	0.048*
Bile duct	9 (15.0%)	77 (19.3%)	0.426
Retroperitoneal tissue	4 (6.7%)	69 (17.3%)	0.088
Lung	17 (28.3%)	100 (25.1%)	0.588
Kidney	7 (11.7%)	44 (11.0%)	0.883
Lymph nodes	22 (36.7%)	188 (47.1%)	0.130
Liver	2 (3.3%)	9 (2.3%)	0.611
Paranasal sinus	16 (26.7%)	114 (28.6%)	0.760
Thyroid	2 (3.3%)	11 (2.8%)	0.802
Pituitary	2 (3.3%)	5 (1.3%)	0.229
Serological features (median, IQR)			
IgG4 (mg/L)	4680 (2195–13,700)	10,000 (4140–19,225)	0.003**
IgG (g/L)	16.87 (13.34–21.06)	19.35 (14.93–25.91)	0.011*
Eosinophils (10^9^/L)	0.15 (0.1–0.29)	0.23 (0.12–0.45)	0.014*
ESR (mm/h)	14 (5.5–41.5)	22 (10–56.4)	0.023*
CRP (mg/L)	1.13 (0.71–5.1)	2.17 (0.78–7.16)	0.081
C3 (g/L)	0.98 (0.74–1.07)	0.93 (0.72–1.11)	0.994
C4 (g/L)	0.18 (0.14–0.24)	0.16 (0.11–0.23)	0.060

IQR represents the interquartile range, and patients’ data that did not meet the normal distribution are shown as median (IQR). SD represents the standard deviation, and the data meeting the normal distribution are shown as mean ± SD

*P < 0.05, **P < 0.01, ***P < 0.001, ****P < 0.0001

### Differences in disease prognosis between the two groups during the follow-up period

**Fig. 4:**
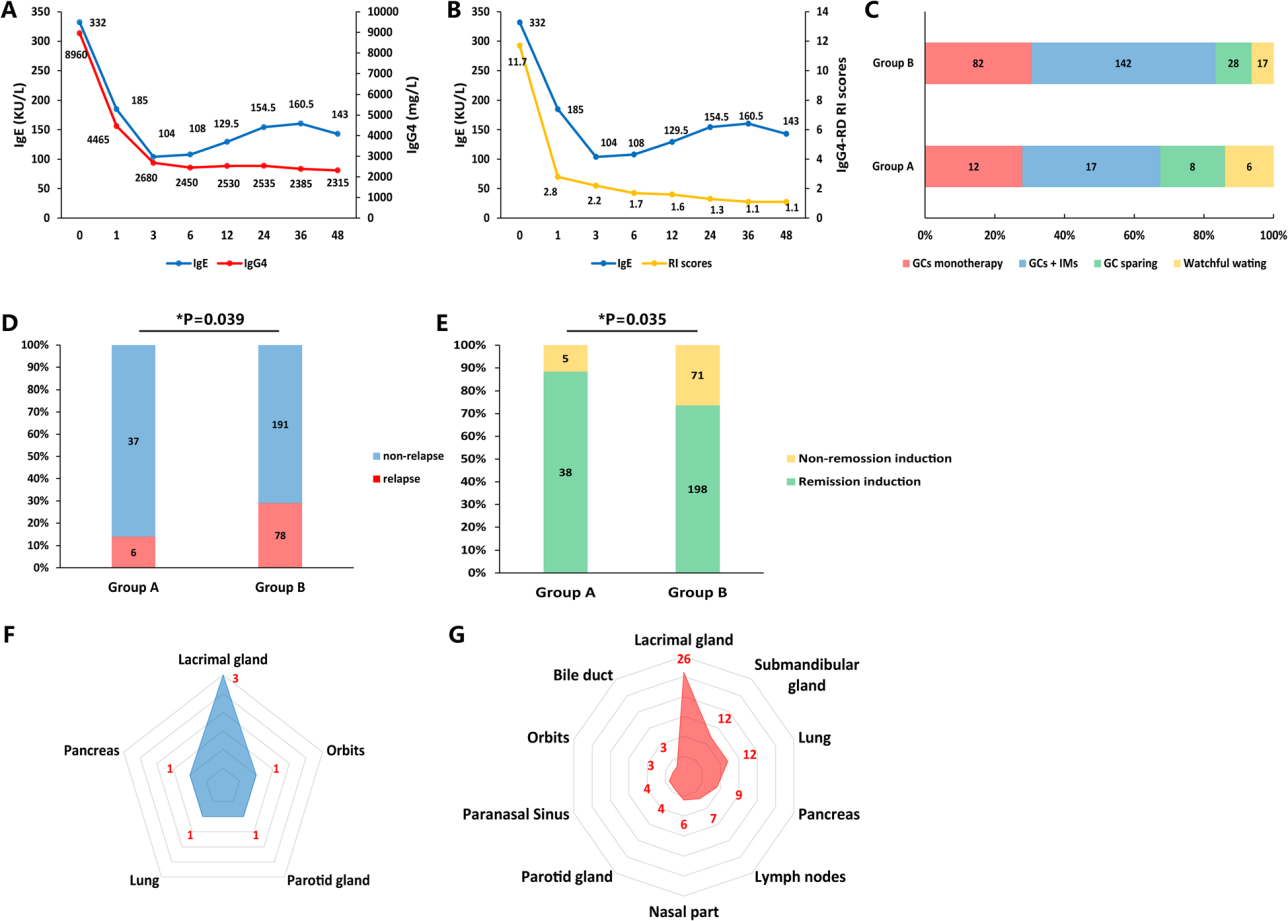
Comparison of disease prognosis between group A and group B patients. **a**, **b** The trends of serum IgE, serum IgG4, and IgG4-RD RI scores are shown as median or mean level changes during the follow-up period. **c** Treatment regimens of the patients involved in the follow-up period. **d**, **e** Comparison of relapse and remission induction between the two groups. **f**, **g** Summary of the relapsed organs between group A (**f**) and group B (**g**) patients (*P < 0.05)

A total of 312 patients (Table 1) who had been followed up for more than 1 year were selected in this study to compare the difference in disease prognosis between group A patients (*n* = 43) and group B patients (*n* = 269). All patients were followed up for a median of 36 (24–54) months. Throughout the follow-up period, patients’ serum IgE levels decreased rapidly and were parallel to the serum IgG4 level and IgG4-RD RI scores in the first 3 months. However, during the long-term follow-up period after the first 3 months, the patients’ serum IgE level showed a slow growth curve and became unparallel to the serum IgG4 level and IgG4-RD RI scores (Fig. 4a, b). We divided patients’ treatment regimens into four types, including GC monotherapy, GCs combined with IM, GCs sparing, and watchful waiting. The treatment regimens between the two groups had no significant statistical difference (Fig. 4c).

Through the chi-square test, we found that group B had more relapsed patients than group A (29.0% vs. 16.2%, *P* = 0.039). Group A had a higher remission induction rate than group B patients (88.4% vs. 73.6%, *P* = 0.035) (Fig. 4d, e). A summary of the patients’ relapsed organs is shown in Fig. 4f, g. Group A patients were more likely to relapse in the lacrimal gland (42.9%), while the lacrimal gland (30.2%), submandibular gland (14.0%), lung (14.0%), and pancreas (10.5%) were the most commonly relapsed organs in group B patients.

### Relationship between serum IgE levels and disease relapse

**Fig. 5:**
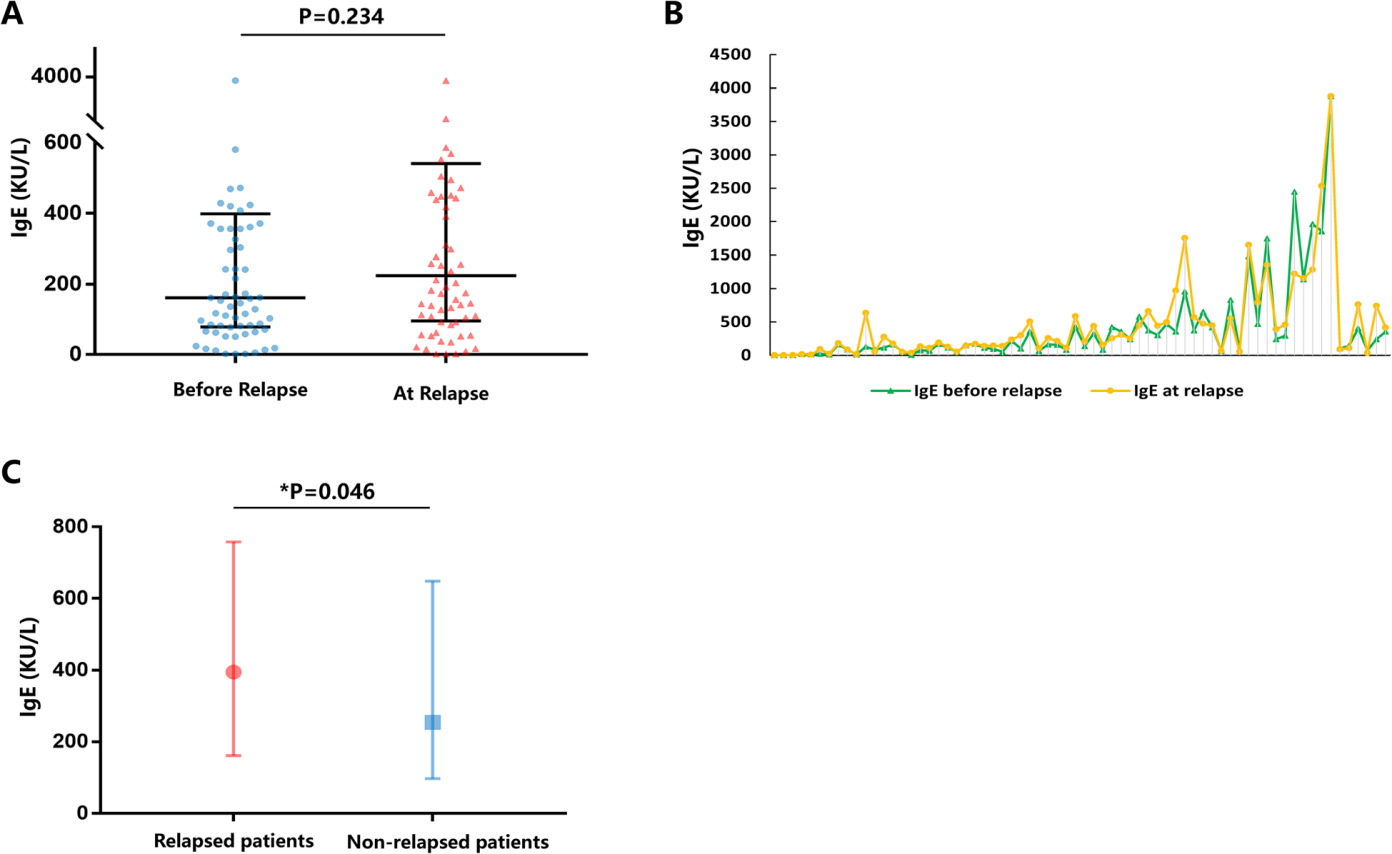
The relationship between serum IgE and disease relapse. **a**, **b** Comparison of the serum IgE levels before and at the disease relapse. **c** Comparison of baseline serum IgE levels between the patients with and without relapse. (In **a**, IgE before relapse level was defined as the serum IgE level during the last follow-up period prior to the disease relapse; IgE at relapse was defined as the serum IgE level at the time of disease relapse. In **b**, the vertical axis represents the IgE levels and the horizontal axis represents every enrolled patient. The two dots on the same vertical dotted line represent IgE levels before and after relapse)

Next, we wanted to determine whether there was a relationship between serum IgE levels and disease relapse. Throughout the whole study, 78 patients experienced disease relapse. We analyzed serum IgE levels before and during patients’ disease relapse. The results showed that the median serum IgE level only showed an increasing trend without any statistical difference at the time of disease relapse (*P* = 0.234, Fig. 5a). In addition, at the time of disease relapse, patients’ serum IgE levels did not positively correlate with relapse; some patients’ serum IgE levels would increase while others showed a decline, indicating that serum IgE levels might not help predict disease relapse during follow-up (Fig. 5b). By comparing the baseline serum IgE levels between patients with or without relapse, we found that relapsed patients had higher serum IgE levels at baseline (*P* = 0.046, Fig. 5c), indicating that high baseline IgE levels were associated with disease relapse during follow-up. Based on the ROC curve, we found that the baseline serum IgE cutoff value for disease relapse was 125 KU/L (sensitivity, 0.845; specificity, 0.694; *P* = 0.037). The univariate Cox regression analysis for all the enrolled patients revealed that serum IgE level > 125 KU/L was related to disease relapse (HR, 2.068 [95% CI 1.144–3.737]; *P* = 0.016). Multivariate analysis found that serum IgE level > 125 KU/L was a risk factor for disease relapse (HR, 1.894 [95% CI 1.022–3.508]; *P* = 0.042; Table 3).

**Table 3:** Univariate and multivariate Cox regression analysis of the relapse factors among the patients in this study

	Univariate analysis		Multivariate analysis	
	HR (95% CI)	*P* value	HR (95% CI)	*P* value
Demographic features				
Age at onset	0.997 (0.980–1.015)	0.767	–	–
Sex (male)	0.941 (0.598–1.483)	0.795	–	–
Disease duration	1.000 (0.994–1.005)	0.920	–	–
Allergy disease	1.223 (0.795–1.879)	0.360	–	–
Number of affected organs	1.015 (0.897–1.148)	0.819	–	–
RI scores	0.991 (0.952–1.030)	0.641	–	–
Serological features				
Serum IgE level > 60 KU/L	2.495 (1.010–6.166)	0.048*	1.643 (0.711–3.798)	0.246
Serum IgE levels (ROC curve)				
≤ 125 KU/L	Ref.	Ref.	–	–
> 125 KU/L	2.068 (1.144–3.737)	0.016*	1.894 (1.022–3.508)	0.042*
Eosinophil count				
≤ 0.5 × 10^9^/L	Ref.	Ref.	–	–
> 0.5 × 10^9^/L	2.389 (1.502–3.799)	< 0.001***	2.354 (1.478–3.750)	< 0.001***
Serum IgG4 level				
≤ 3 ULN	Ref.	Ref.	–	–
3 ULN < IgG4 ≤ 5 ULN	0.757 (0.348–1.644)	0.482	–	–
> 5 ULN	1.158 (0.704–1.904)	0.563	–	–
Organ involvements				
Lacrimal gland	1.832 (1.171–2.865)	0.008**	1.732 (1.053–2.849)	0.031*
Submandibular gland	1.591 (1.021–2.480)	0.040*	1.097 (0.671–1.793)	0.711
Parotid gland	1.134 (0.657–1.957)	0.652	–	–
Pancreas	1.562 (0.976–2.501)	0.063	–	–
Bile duct	1.384 (0.791–2.241)	0.254	–	–
Lung	1.258 (0.788–2.008)	0.337	–	–
Kidney	2.937 (1.075–8.023)	0.036*	2.793 (1.019–7.659)	0.046*

HR represents the hazard ratio. 95% CI represents the 95% confidence interval. ULN refers to the upper limit of normal

*P < 0.05, **P < 0.01, ***P < 0.001

### Differences in the risk factors for relapse between the two groups

**Fig. 6:**
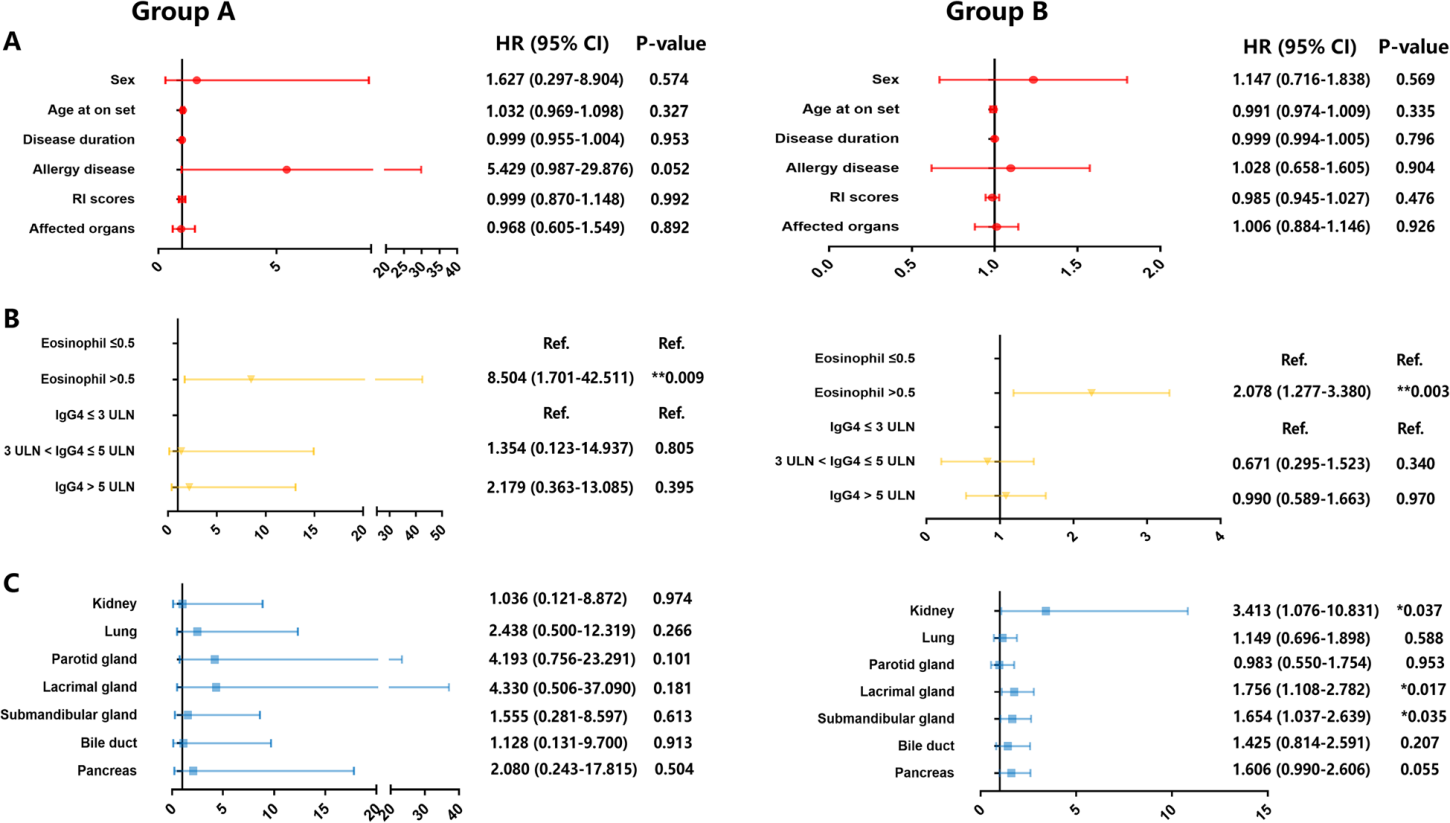
Cox univariate regression analysis of the two groups. **a** Cox regression analysis for the demographic features. **b** Cox regression analysis for the serological features. **c** Cox regression for the organ involvement (HR represents the hazard ratio, 95% CI represents the 95% confidence interval, ULN refers to the upper limit of normal (serum IgG4, 1350 mg/L). The unit for eosinophil count was 10^9^/L)

Since the two groups had different clinical features and relapse rates during the follow-up period, we wanted to know whether they had different risk factors for disease relapse. The results of the Cox regression analysis are shown in Fig. 6. Through the Cox univariate regression analysis, we found that there were no demographic features as risk factors for relapse in both groups, while the elevation of the eosinophils was a risk factor for relapse in both groups A (HR, 8.504 [95% CI 1.071–42.511]; *P* = 0.009) and group B (HR, 2.078 [95% CI 1.277–3.380]; *P* = 0.003). Then through the Kaplan-Meier curve, we found that serum IgG4 level > 10 ULN (13,500 mg/L) at baseline was also correlated with disease relapse (HR, 2.021 [95% CI 1.11–3.679]; *P* = 0.021, supplementary figure 1), just like our previous risk factor study. The biggest differences of risk factors for the two groups were the affected organs, for we did not find any affected organ as a risk factor for relapse in group A patients, while the involvement of the lacrimal gland (HR, 1.756 [95% CI 1.108–2.782]; *P* = 0.017), submandibular gland (HR, 1.654 [95% CI 1.037–2.639]; *P* = 0.035), and kidney (HR, 3.413 [95% CI 1.076–10.831]; *P* = 0.037) were the risk factors for group B patients.

## Discussion

Elevated serum IgE levels were observed in 35.0–88.9% of IgG4-RD patients in previous studies [5, 6, 13, 14]. It has already been well acknowledged that the Tfh immune response is a key pathway for the production of both IgG4 and IgE [3, 4, 15]. However, the serum IgE level varies in a wide range in newly diagnosed IgG4-RD patients [13, 14, 16], and there is still uncertainty about the role of serum IgE in IgG4-RD patients. To our knowledge, this study is the largest cohort study reporting the relationship between serum IgE levels and clinical features, as well as the outcomes of IgG4-RD patients.

Our study showed that the baseline serum IgE level was positively correlated with the serum IgG4 level and eosinophil count, which is consistent with the results of previous studies [5, 6]. Nishida et al. [17] analyzed the lymphoid tissue of 23 IgG4-related lymphadenopathy patients and found abundant mast cells with strong cytoplasmic staining for IgE and high-affinity IgE receptor immunohistologically. IgE production was likely driven by IL-4 and IL-13 in previous studies [18, 19]. Some cytokines were thought to be the major regulators of IgG-subclass responses. Akiyama et al. reported that IL-4 and IL-21 released by Tfh cells could increase class switch (IgG to IgG4) in IgG4-RD patients and also induce the differentiation of plasmablasts and IgG4 production [20]. In addition, IL-4 was reported to promote IgG1/IgE class switching in B cells [21]. Thus, the correlation between serum IgE levels and serum IgG and IgG4 levels found in our study implied the potential role of class switching mechanism and IgE in the development of IgG4-RD. Therefore, it is interesting to investigate the correlations between Tfh-related serum cytokine levels and IgE, as well as to compare the IgE level immunohistologically, between groups A and B, which may shed light on the role of IgE in the pathogenesis of IgG4-RD in future studies.

Nearly half of the patients in our cohort had a history of allergic disease, particularly allergic rhinitis, which is also supported by reports from several studies [6, 21, 22]. Moreover, there was an association between allergic rhinitis and IgG4-RD head and neck phenotypes [23, 24]. The overall incidence of allergic rhinitis and asthma in our cohort was slightly higher than that in normal adult populations in China (21.6% vs. 17.6% and 4.4% vs. 2.46%, respectively) [25, 26]. The relationship between IgE and allergy in IgG4-RD remains controversial. Della Torre et al. and Culver et al. [5, 6] reported that IgG4-RD patients with a history of allergic disease had higher serum IgE levels than those without, and further study showed that Th2 memory cells were largely restricted to IgG4-RD patients with allergies [27]. However, no significant difference was found in the serum IgE levels between allergic and non-allergic patients in a large cohort from Italy and in Japanese IgG4-RD patients [21, 28]. To date, our study is the largest cohort evaluating the relationship between serum IgE and a history of allergy in IgG4-RD patients, and we found that group B had more patients with allergy disease than group A. Our study also showed that group B had more organ involvement and higher IgG4-RD RI scores than group A, indicating that patients with high baseline IgE levels might have higher disease activity.

Wallace et al. and our previous study [7, 29] both reported that baseline elevations in serum IgG4 levels were risk factors for IgG4-RD relapse; the present study also had the similar results in patients with GC monotherapy and GCs + IM therapy (Supplementary figure 1). Other studies [6, 7] showed that baseline serum IgE level was a predictor of relapse of IgG4-RDs. In our study, serum IgE level > 125 KU/L was a risk factor for disease relapse. We also found that group A patients had a higher remission induction rate than group B patients. Moreover, our previous study reported that during the follow-up period, re-elevation of serum IgG4 levels could also help in predicting the relapse of IgG4-RD [29]. However, unlike serum IgG4, the change in serum IgE level did not positively correlate with disease relapse in this study. Serum IgE levels decreased rapidly in the first 3 months after treatment and were also parallel to the serum IgG4 level and IgG4-RD RI scores in the first 3 months, which was consistent with several studies published before [6, 30], indicating that serum IgE level is correlated with response to therapy in the early stage. However, during the long-term follow-up period after the first 3 months, the patient’s serum IgE level had a slow growth curve and became unparallel to the serum IgG4 level and IgG4-RD RI scores. The trends of serum IgE reflected the complexity of IgE in the pathogenesis of IgG4-RD.

Another cohort study from China reported that affected lacrimal and parotid glands were more common in patients with relapse in the full spectrum of IgG4-RD patients [31]. In this study, we found that an elevated eosinophil count was a risk factor for relapse in both groups, which was also reported by Wallace et al. [7]. In group B patients, involvement of the lacrimal gland, submandibular gland, and kidney were risk factors for disease relapse. There were no other risk factors for relapse in group A patients, which might be due to the relatively small sample size of group A in this study.

Our study was based on one of the largest cohorts of Chinese IgG4-RD patients and showed the relationship between serum IgE levels and disease activity and relapse in Chinese patients. However, there are a few limitations to our study. The reference values of serum IgE test might vary among people of different ethnicities and ages, and the normal range of serum IgE in our study was based on the range used in our institution. Larger prospective cohorts are needed to determine an optimal cutoff value of serum IgE level on the ROC curve. Asian and non-Asian cases have different spectra of organ involvement [32]; therefore, a multicenter study including patients with more diverse ethnicities might help us better understand the disease.

## Conclusion

In conclusion, serum IgE levels were commonly elevated in newly diagnosed IgG4-RD patients and positively correlated with serum IgG4 levels and peripheral eosinophil counts. IgG4-RD patients with high serum IgE levels at baseline were more likely to have higher disease activity, and baseline high IgE levels were associated with disease relapse.

## Supplementary information

**Additional file 1: Supplementary Figure 1.** The Kaplan-Meier curve for the IgG4-RD patients (GCs monotherapy and GCs + IMs therapy) in different serum IgG4 levels groups.

## Data Availability

The datasets used and/or analyzed during the current study are available from the corresponding author upon reasonable request.
