# Supplementary material for: Serum IgE in the clinical features and disease outcomes of IgG4-related disease: a large retrospective cohort study
Source: Arthritis Res Ther. 2020 Oct 23;22:255. doi: 10.1186/s13075-020-02338-1 (PMC7583198; doi:10.1186/s13075-020-02338-1)
Supplement: Supplementary file 1 — Additional file 1: Supplementary Figure 1. The Kaplan-Meier curve for the IgG4-RD patients (GCs monotherapy and GCs + IMs therapy) in different serum IgG4 levels groups. [file 13075_2020_2338_MOESM1_ESM.docx]

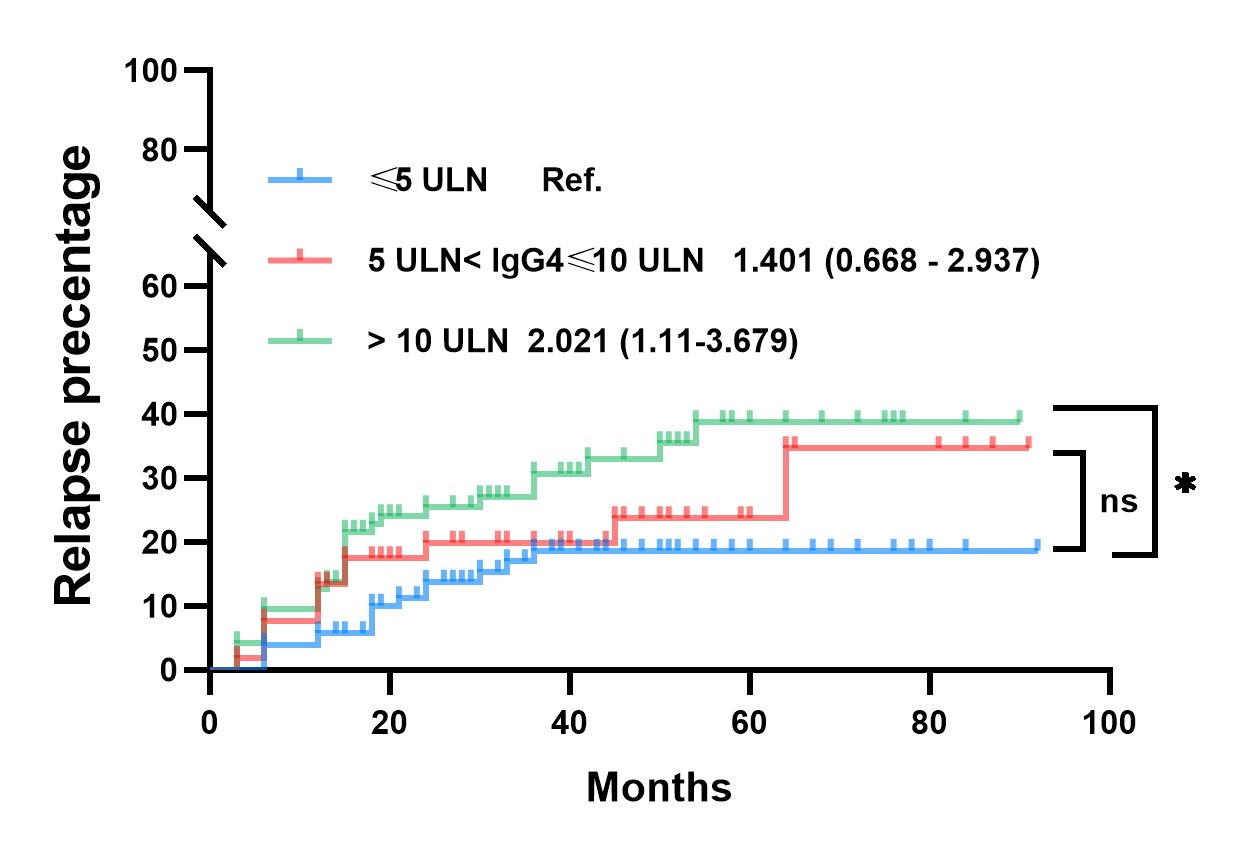


Supplementary Figure 1. The Kaplan-Meier curve for the IgG4-RD patients (GCs monotherapy and GCs + IMs therapy) in different serum IgG4 levels groups. ULN represented for the upper limit of normal.
